# Evaluating the involvement of autolysosomes in the nuclear translocation of fluorescent proteins

**DOI:** 10.1002/2211-5463.70279

**Published:** 2026-06-03

**Authors:** Keiichi Ikeda

**Affiliations:** ^1^ Core Research Facilities for Basic Science, Research Centre for Medical Sciences The Jikei University School of Medicine Tokyo Japan

**Keywords:** autophagosome, EGFP, lysosome, mCherry, nucleocytoplasmic transport, protein tracking

## Abstract

During the construction of control mCherry‐labelled HeLa cells, we unexpectedly observed red fluorescence in cell nuclei and therefore investigated the mechanisms underlying the transport of fluorescent proteins (FPs) into the nuclei. We confirmed that mCherry and mCherry/EGFP tandem FPs were translocated into the nucleus and found that FPs are taken up by autophagosomes and translocated into the nucleus after entering lysosomes. However, pharmacological inhibition of autophagosome–lysosome fusion and syntaxin 17 knockdown decreased the nuclear translocation of mCherry in HeLa cells, but not in HepG2 cells, indicating that autophagy may also be involved in the nuclear translocation of FPs. Electron microscopy revealed that autolysosomes fused with the nuclear envelope and continued into the nucleus in HeLa cells but not in HepG2 cells, indicating that autophagy is involved in the nuclear translocation of FPs in HeLa cells. In addition, immunotransmission electron microscopy revealed that FPs can be transported directly into the nucleus through the nuclear pore complex. Our results suggest that autophagy is involved in the intracellular degradation and nuclear translocalisation of FPs.

## Graphical abstract

Endogenously expressed fluorescent proteins can be degraded by autophagy and transported to cell nuclei via the nuclear pore complex. But in some cell lines, for example, HeLa cells which are positive for immunoreactivity of a receptor ligand, such as UCN I, in cell nuclei, fusion of autolysosome with the nuclear envelope is involved in the nuclear localization of endogenously expressed fluorescent proteins.

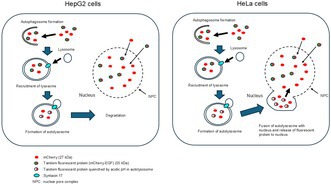

**Abbreviations**
BSA: bovine serum albumin
DMSO: dimethyl sulfoxide
EACC: ethyl (2‐(5‐nitrothiophene‐2‐carboxamido) thiophene‐3‐carbonyl) carbamate
E‐MEM: Eagle's minimum essential medium
FBS: fetal bovine serum
FP: fluorescent protein
ITEM: immunotransmission electron microscopy
LC3B: microtubule‐associated protein 1 light chain 3B
NE: nuclear envelope
NLS: nuclear localization signal
NPC: nuclear pore complex
NSF: soluble N‐ethylmaleimide‐sensitive factor
PB: phosphate buffer
PFA: paraformaldehyde
SD: standard deviation
TBS: Tris‐buffered saline
TEM: transmission electron microscopy
UCN: urocortin

Some receptors localize in the nucleus along with their ligands, such as urocortin (UCN) I [1], angiotensin II [2, 3, 4], nerve growth factor β [5], interleukin 1 [6], insulin [7, 8], norepinephrine [9], and growth hormone [10, 11, 12]. However, the detailed mechanisms underlying this process remain unclear. Therefore, we are investigating the nuclear transport mechanisms of such receptor ligands, especially corticotropin‐releasing factor receptors (i.e., CRF_1_ and CRF_2_), receptors for UCN I [13], but we unexpectedly found that the HeLa cells which were positive for nuclear UCN I immunoreactivity (unpublished data) were emitting red fluorescence (mCherry) in the nuclei (see supplemental methods and Fig. S1) during the generation of control red fluorescent HeLa cells transfected with the pmCherry‐C1 plasmid (Clontech Laboratories, Inc., Mountain View, CA, USA) for imaging using ArrayScan XT (Thermo Fischer Scientific, Inc., Waltham, MA, USA). Same results were obtained with HeLa cells transfected with the pAcGFP‐C1 plasmid (Clontech Laboratories, Inc.; Fig. S1). These results suggest that the mechanisms that transport the endocytosed receptor ligands and endogenously synthesized proteins into the cell nucleus may exist; however, the underlying mechanisms remain ambiguous. It was reported that several proteins less than 30–60 kDa could be transported via the nuclear pore complex (NPC) [14, 15, 16]. But protein aggregates could be degraded via aggrephagy, a type of autophagy [17] and another intracellular route of degradation process. In addition, fluorescent proteins (FPs) may possibly be degraded in an autophagy‐related mechanism [18]. Therefore, we hypothesized that FPs can be not only transported into the nucleus via NPC but also degraded  autophagy, which is possibly involved in their nuclear translocation in HeLa cells, and it should be confirmed whether the lysosome is involved in the degradation of FPs and the autophagosome is involved in the nuclear translocalization of FPs as an entrance of the lysosome system. In this study, we aimed to elucidate the intracellular mechanisms of protein transport into the nucleus.

## Materials and methods

### Cell culture

HeLa cells (RRID: CVCL_0030, American Type Culture Collection [ATCC], Manassas, VA, USA) showing nuclear FP immunoreactivity and HepG2 cells (ATCC HB‐8065, original source is ATCC, provided from Prof. Akihito Tsubota, Project Research Unit, The Jikei University School of Medicine) which were negative for nuclear UCN I (unpublished our data) were cultured in the Eagle's minimum essential medium (E‐MEM, Fujifilm Wako Pure Chemical Co., Osaka, Japan, or Minimal Essential Medium, Merck KGaA, Darmstadt, Germany) supplemented with 10% fetal bovine serum (FBS, Serana Europe GmbH, Brandenburg, Germany and MP Biomedicals, Inc., Irvine, CA, USA), 1% nonessential amino acids (Thermo Fischer Scientific Inc. or Fujifilm Wako Pure Chemical Co.), and 1% GlutaMAX Supplement (Thermo Fischer Scientific Inc.).

HepG2 and HeLa cells have been authenticated by the records of usage in our laboratory in the past three years. In addition, all experiments were performed with mycoplasma‐free cells.

### Transfection with pmCherry‐C1 and pAcGFP‐C1 plasmid

Transfection with pmCherry‐C1 and pAcGFP‐C1 plasmids was performed as previously described [19]. Briefly, 1.0 μg of plasmid was added to 1.0 × 10^5^ HeLa cells, along with 2 μL FuGENE HD (Promega Co., Madison, WI, USA). Forty‐eight hours post transfection, cells were selected using 500 μg·mL^−1^ G418 disulfate (InvivoGen, San Diego, CA, USA) for 2 weeks [20].

### Imaging of HeLa cells transfected with pmCherry‐C1 and pAcGFP plasmid

Cells stably expressing fluorescent protein were propagated and seeded in a 35‐mm glass bottom dish (φ27mm; AGC Techno Glass Co., Ltd., Haibara‐gun, Shizuoka prefecture, Japan) at a density of 2.0 × 10^5^ cells per dish and incubated overnight at 37 °C in a 5% CO_2_ humidified atmosphere. The following day, the cells were washed with 1× Dulbecco's phosphate‐buffered saline (DPBS; 10‐fold dilution of 10× DPBS; Sigma‐Aldrich Co., St. Louis, MO, USA) and fixed in 4% paraformaldehyde (PFA; Fujifilm Wako Pure Chemical Co., Osaka, Japan) for 10 min. Cells were stored in 0.1 mol·L^−1^ phosphate buffer containing 0.1% PFA until imaging. In addition, the cell nuclei were stained with NucBlue Live ReadyProbes (Thermo Fischer Scientific, Inc.) according to the manufacturer's protocol immediately before imaging. Cells were subjected to imaging using an LSM‐880 confocal laser microscope (Carl Zeiss AG, Jena, Germany).

### Plasmid construction, transfection, and selection of the cells stably expressing fluorescent protein

mCherry‐expressing (pmCherry Puro plasmid, VB900088‐2488xwp, pRP[Exp]‐Puro‐EF1A>mCherry) and mCherry‐EGFP tandem FP‐expressing (pTandem Puro plasmid, VB240315‐1206ypf, pRP[Exp]‐Puro‐EF1A>mCherry/3xGGGGS/EGFP) were designed and constructed by VectorBuilder Inc. (Chicago, IL, USA). The sequences and vector maps of the plasmids used in this study are available at www.vectorbuilder.com. Molecular weights of mCherry and Tandem FP were estimated to be about 27 kDa and 55 kDa, respectively (calculated by https://web.expasy.org/compute_pi/).

Both HepG2 and HeLa cells were transfected with these plasmids as previously described [19]. Briefly, 1 μg of plasmid was applied per 1.0 × 10^5^ target cells using 2 μL of FuGENE HD transfection reagent (DNA:FuGENE HD = 1 : 2), and the cells were seeded in a 12‐well plate at a density of 0.5 × 10^5^ cells/well. At 48 h post transfection, HeLa cells stably expressing FPs (mCherry and tandem FPs) were selected using the E‐MEM supplemented with 10% FBS and 2 μg of puromycin (Nacalai Tesque, Inc., Kyoto, Japan) for two weeks, as previously described [21, 22]. HeLa cells positive for nuclear FPs were selected via single‐cell cloning. On the other hand, transfected HepG2 cells (plasmid expressing mCherry and tandem FPs) were applied to select with E‐MEM containing 10% FBS and 8 μg puromycin [23] (InvivoGen) since 72 h after transfection. After culturing in E‐MEM containing 10% FBS and 8 μg puromycin for 1 week, a fluorescent‐protein‐positive HepG2 cell was further selected by single cell cloning.

### Western blot analysis of fluorescent proteins (FPs) expressing in HepG2 and HeLa cells

To confirm the molecular weight of expressed FPs, HepG2 and HeLa cells expressing FPs were propagated in T75 flasks and harvested with M‐PER Mammalian Protein Extraction Reagent (Thermo Fischer Scientific Inc.) according to manufacturer's protocol after the cells reached subconfluence. Protein contents of cell samples were measured by BCA Protein Assay Reagent Kit (Thermo Fischer Scientific Inc.). Western blotting analysis were performed after proteins were electrophoresed in Tris‐sodium dodecyl sulfate‐glycine buffer (Tris: 25 mmol·L^−1^, sodium dodecyl sulfate: 0.1%, glycine: 192 mmol·L^−1^). Briefly, 10 μg of protein samples was applied to 10% Mini‐PROTEAN TGX Precast Protein Gels (Bio‐Rad Laboratories, Inc., Hercules, CA, USA) after denatured with EzApply (Atto Co., Ltd., Tokyo, Japan) and electrophoresed with molecular markers (Precision Plus Protein WesternC Standards, Bio‐Rad Laboratories, Inc.). Proteins were blotted onto poly vinylidene di‐fluoride (PVDF) membranes (Trans‐Blot Turbo Mini 0.2 μm PVDF Transfer Packs, Bio‐Rad Laboratories, Inc.) by Trans‐Blot Turbo Transfer system (Bio‐Rad Laboratories, Inc.) according to manufacturer's protocol (Mixed Mw). Blotted PVDF membranes were blocked with TBS containing 0.05% polysorbate (10 times dilution of 10× Tris Buffered Saline with Tween 20, Santa Cruz Biotechnology, Inc., Santa Cruz, CA, USA) and 3% bovine serum albumin (Nacalai tesque, Inc.) for an hour. Then, anti‐mCherry antibody (rabbit IgG, Proteintech Group, Inc., Rosemont, IL, USA [1 : 1000]) were applied to blotted membrane and incubated at room temperature (RT, 20–25 °C) for 1 and half hours. Thereafter, membranes were subjected to secondary antibodies (anti‐rabbit IgG‐Peroxidase antibody [1 : 20 000], A0545, Sigma‐Aldrich Co.) and Precision Protein StrepTactin‐HRP Conjugate (1 : 50 000, Bio‐Rad Laboratories, Inc.) and incubated at RT for an hour. Finally, membranes were incubated with Pierce ECL Plus Western Blotting Substrate (Thermo Fischer Scientific Inc.) for 5 min at RT and visualized by ChemiDoc Touch Imaging system (Bio‐Rad Laboratories, Inc.).

### Fluorescence imaging of HepG2 and HeLa cells with mCherry and tandem FP by laser confocal microscopy

Live cell imaging was performed to clarify the nuclear translocation mechanism of mCherry. Briefly, HeLa cells transfected with pmCherry Puro and seeded in 35‐mm glass bottom dishes (φ27 mm; AGC Techno Glass Co., Ltd., Haibara‐gun, Shizuoka prefecture, Japan) at a density of 4.0 × 10^5^ cells per dish and incubated in E‐MEM containing 10% FBS overnight at 37 °C in a 5% CO_2_ humidified atmosphere. On the next day, the cells were stained with or without LysoTracker Deep Red (final concentration: 60 nmol·L^−1^; Thermo Fischer Scientific, Inc.) and DAPGreen—Autophagy Detection (final concentration: 0.1 μmol·L^−1^; Dojindo Laboratories, Mashikimachi, Kumamoto Prefecture, Japan) [24]. Equivalent doses of dimethyl sulfoxide (DMSO; Nacalai Tesque Inc.) were added to the vehicle cultures.

HepG2 and HeLa cells transfected with the pTandem (mCherry/EGFP) Puro plasmid were used for confocal laser imaging with or without LysoTracker Deep Red after seeding in 35‐mm glass bottom dishes, as described above. Then, cell nuclei were stained with NucBlue Live ReadyProbes immediately before imaging, according to the manufacturer's protocol. Finally, live cell (time‐lapse) images were acquired at 20‐s intervals via LSM‐880 laser confocal microscopy at 37 °C in a 5% CO_2_ humidified atmosphere.

And to confirm the colocalization of autophagosome and mCherry because the previous report demonstrated that GFP‐like protein entered the lysosome via an autophagy‐related mechanism [18], HeLa cells overexpressing mCherry were applied for colocalization study with autophagosomal membrane protein, microtubule‐associated protein 1 light chain 3B (LC3B) [25, 26, 27]. Briefly, HepG2 and HeLa cells expressing mCherry were seeded in a 35‐mm glass bottom dish at a density of 3.0 × 10^5^ cells per dish and incubated overnight at 37 °C in a 5% CO_2_ humidified atmosphere. Next day, HeLa cells overexpressing mCherry were infected with Premo Autophagy Sensor LC3B‐GFP (30 particles per plated cells, Premo Autophagy Sensor LC3B‐GFP kit [BacMam 2.0], P36235, Thermo Fischer Scientific Inc.), a marker for autophagosome [25, 26, 27], according to the manufacturer's recommendation. Then, cell nuclei were stained with NucBlue Live ReadyProbes and LysoTracker Deep Red immediately before imaging and subjected to live cell imaging. Live cell images were acquired at 37 °C in a 5% CO_2_‐humidified atmosphere via LSM‐880 laser confocal microscope.

In addition to live cell imaging, laser confocal microscopy was performed in fixed samples of HepG2 and HeLa cells to confirm differential degradation of fluorescent protein because fluorescence of EGFP could be quenched by acidic pH and degraded in lysosome easier than mCherry [28]. HepG2 and HeLa cells transfected with pTandem Puro plasmid were seeded in a 35‐mm glass bottom dish at a density of 4.0 × 10^5^ cells per dish and incubated overnight at 37 °C in a 5% CO_2_ humidified atmosphere. The following day, cells were washed with 1× DPBS and fixed in 4% PFA for 10 min. Then, cells were stored in 0.1 mol·L^−1^ phosphate buffer containing 0.1% PFA until imaging (HepG2 cells: 4 days, HeLa cells: 3 days). Nuclei were stained with NucBlue Live ReadyProbes, according to the manufacturer's protocol, immediately before imaging. After labelling with NucBlue Live ReadyProbes, fixed cells were washed with 1× TBS and stored in TBS. Then, cells were subjected to imaging using LSM‐880 confocal laser microscope.

To confirm the nuclear translocation route of FPs, we tested whether the fluorescence of mCherry in the cell nuclei of HepG2 and HeLa cells is decreased by the autophagosome inhibitor, ethyl (2‐[5‐nitrothiophene‐2‐carboxamido] thiophene‐3‐carbonyl) carbamate (EACC; TargetMol Chemicals Inc., Boston, MA, USA) [29]. Briefly, HeLa cells transfected with pmCherry Puro plasmid were seeded in a 6‐well EZVIEW Glass Bottom Culture Plate LB (AGC Techno Glass Co., Ltd.) at a density of 4.0 × 10^5^ cells per well and incubated in E‐MEM containing 10% FBS overnight at 37 °C in a 5% CO_2_ humidified atmosphere. On the next day, the cells were stimulated with equivalent doses of DMSO (0.1%) or 10 μmol·L^−1^ EACC [29] for 1, 3, 6, and 24 h, fixed with 4% PFA, and stored in 0.1 mol·L^−1^ phosphate buffer containing 0.1% PFA until imaging. Subsequently, cell nuclei were stained with NucBlue Live ReadyProbes, according to the manufacturer's protocol immediately before imaging using the LSM880 laser confocal microscope (6‐well glass bottom plate).

### Excitation and detection conditions of laser confocal microscopy

Laser settings for confocal laser microscopy were as follows: blue fluorescence with an excitation wavelength of 405 nm and a detection window of 410–508 nm, green fluorescence with an excitation wavelength of 488 nm and a detection window of 493–594 nm, red fluorescence with an excitation wavelength of 561 nm and a detection window of 569–649 nm, and infrared fluorescence with an excitation wavelength of 633 nm and a detection window of 638–747 nm. The acquired images were processed using the ZEN software (blue and black editions; Carl Zeiss Microscopy GmbH, Jena, Germany).

### Quantitative analysis of pharmacological inhibition and RNA interference of syntaxin 17 on the nuclear translocation of mCherry

To confirm the effects of the pharmacological effects of EACC quantitatively, HepG2 and HeLa cells stably expressing mCherry were seeded in a 96‐well optical plate at a density of 2.0 × 10^4^ per well. The following day, cells were treated with or without EACC for 3 h. The relative intensity of nuclear mCherry in HeLa cells stimulated by EACC was normalized by that stimulated by dose‐matched DMSO. After treatment with EACC for 3 h, cells were fixed with 4% PFA as described above and stored in 0.1 mol/L PB containing 0.1% PFA until quantification. On the day of image acquisition, nuclei were stained with NucBlue Live ReadyProbes, according to the manufacturer's protocol and finally stored in 1× TBS.

In addition to pharmacological inhibition of syntaxin 17, which is required to fuse autophagosome with lysosome [30], RNA interference of syntaxin 17 was also performed to confirm the involvement of the fusion of autophagosome in nuclear translocalization of FPs. RNA interference of syntaxin 17 in HepG2 cells and HeLa cells was performed to confirm the effects of EACC on nuclear translocation of mCherry using STX17 siRNA (*Silencer* Selelct, a29992; Thermo Fischer Scientific Inc.) because the fusion of autophagosome and lysosome is required of syntaxin 17. HepG2 cells and HeLa cells were seeded in a 96‐well optical plate at a density of 5000 cells per well and treated with STX17 siRNA using Lipofectamine RNAi MAX (Thermo Fischer Scientific Inc.) according to the manufacturer's protocol (reverse transfection protocol). *Silencer* Select Negative Control No. 2 siRNA (Thermo Fischer Scientific Inc.) was used as a control siRNA and culture medium was changed to E‐MEM containing 10% FBS at 6 h after transfection. Furthermore, cells were cultured in E‐MEM containing 10% FBS for 3 days [30].

After washing with 1× DPBS and fixed with 4% PFA (if necessary, cells were stored in 0.1 mol·L^−1^ PB containing 0.1% PFA until imaging) and cell nuclei were stained with NucBlue Live ReadyProbes and stored in 1 × TBS, images were acquired by BZ‐X800 (magnitude of objective lens was 10×, Keyence Co., Osaka, Japan) and analysed the colocalization of Heochst 33 342 and mCherry in 3 to 4 wells per group by BZ X800 Analyzer (Keyence Co.). Average intensity was measured, and mean intensity of treatment group was calculated as follows:

Total intensity/nuclei/well=total mCherry colocalized with blue fluorescence/number of nuclei

$$\begin{array}{l} \text{Mean intensity of mCherry colocalized}/\text{group}\\ \;=\sum \text{Total intensity}/\text{number of nuclei}\;\left(n=3\;\text{to}\;4/\text{group}\right) \end{array}$$

Data were represented as mean (SD). Statistical analysis was performed by Dunnett's test (BellCurve for Excel, Social Survey Research Information Co., Ltd., Tokyo, Japan). Data were represented as mean (standard deviation [SD]) and *P* value < 0.05 was considered to be significant.

### Electron microscopy of HeLa cells stably expressing mCherry

To confirm fusion of lysosome and nucleus, HepG2 and HeLa cells stably expressing mCherry were harvested by trypsinization. After cells were washed twice with 1× DPBS, cells were fixed with 2% glutaraldehyde (Nacali tesque, Inc.) for more than 30 min. Cells were washed with 0.1 mol·L^−1^ PB containing 8% sucrose three times and treated with 0.1 mol/L PB containing 1% OsO_4_ at 4 °C for 1 h. And cells were dehydrated with ethanol gradient (50% to 100%). Thereafter, cells were embedded in epoxy resin (Epok 812; Okenshoji Co., Ltd., Tokyo, Japan). Cells were sliced to ultrathin section and mounted on to grid. Finally, cells were stained with the saturated acetate salt of uranium oxide and Reynolds lead solutions. Then, stained cells were subjected to transmission electron microscopy (TEM, JEM‐1400Plus; JEOL Ltd, Akishima City, Tokyo, Japan).

### Immunoelectron microscopy of HepG2 cells expressing tandem FP

To investigate whether FPs are transported through NPCs, ITEM was performed using HepG2 cells transfected with or without pTandem Puro plasmid. Briefly, cells were seeded in a 96‐well plate in which wells Au (24 K)‐discs were placed (1 disc per well) prior to cell seeding, at a density of 2.0 × 10^4^ cells per well. Two days after seeding, cells were rapidly frozen in liquid propane (−175 °C) and fixed. Cells were treated with 2% tannic acid in ethanol containing 2% distilled water at −80 °C for 48 h, followed by further treatment at −20 °C for 3 h and 4 °C for 1 h. Cells were dehydrated in anhydrous ethanol at 4°C overnight. Cells were embedded in LR white (London Resin Co., Ltd., Berkshire, UK). Briefly, cells were immersed in the solution consisting of LR white and ethanol (1 : 1) at 4 °C for 30 min, followed by the treatment of LR white at 4 °C for 30 min, three times. Thereafter, cells were embedded in LR white and polymerized at 50 °C overnight. Ultrathin sections (80 nm) were generated and mounted onto a Ni grid. Cells were treated with primary antibody for GFP (50×; Anti‐GFP antibody, ab6556, Abcam limited, Cambridge, UK) at 4 °C overnight, followed by treatment with secondary antibody, a goat anti‐rabbit IgG (20×, 15 nm Au colloid conjugated, BBI Solutions, Crumlin, UK) at RT for 2 h. Antibodies were dissolved in phosphate buffered saline containing 1% BSA and 1.5% goat normal serum (Vector Laboratories, Inc., Newark, CA, USA). Finally, cells were stained with 2% uranyl acetate at RT for 5 min and Lead stain solutions (Sigma‐Aldrich Co.) at RT for 3 min. Stained cells were subjected to transmission electron microscopy (JEM‐1400Plus, JEOL Ltd.) for acquisition of images of HepG2 cells transfected with or without pTandem Puro plasmid.

## Results

### Confirmation of molecular size of FPs expressing in HepG2 and HeLa cells

Western blotting analysis revealed that molecular sizes of FPs expressing in HepG2 and HeLa cells were corresponding to expected molecular size, indicating that it is unlikely that amino acid sequences including the known nuclear localization signal (NLS) [31] were added to expressing FPs (Fig. S2).

### Tracking of expressing fluorescent protein, autophagosome and lysosome

#### Tracking of FPs in HepG2 and HeLa cells accompanied with autophagosome, lysosome, and nucleus

**Fig. 1:**
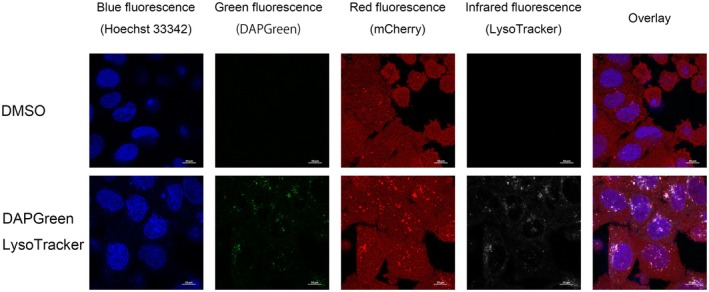
HeLa cells transfected with the pmCherry Puro plasmid were stained with (lower image) or without (upper image) DAPGreen and LysoTracker Deep Red. The magnification of the objective lens was 100×. Scale bar, 10 μm.

**Fig. 2:**
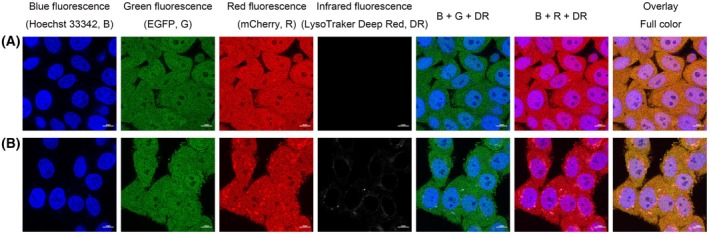
HeLa cells transfected with the pTandem (mCherry/EGFP) Puro plasmid were stained with dimethyl sulfoxide (DMSO; A) and LysoTracker Deep Red (B). The magnification of the objective lens was 100×. Scale bar, 10 μm.

**Fig. 3:**
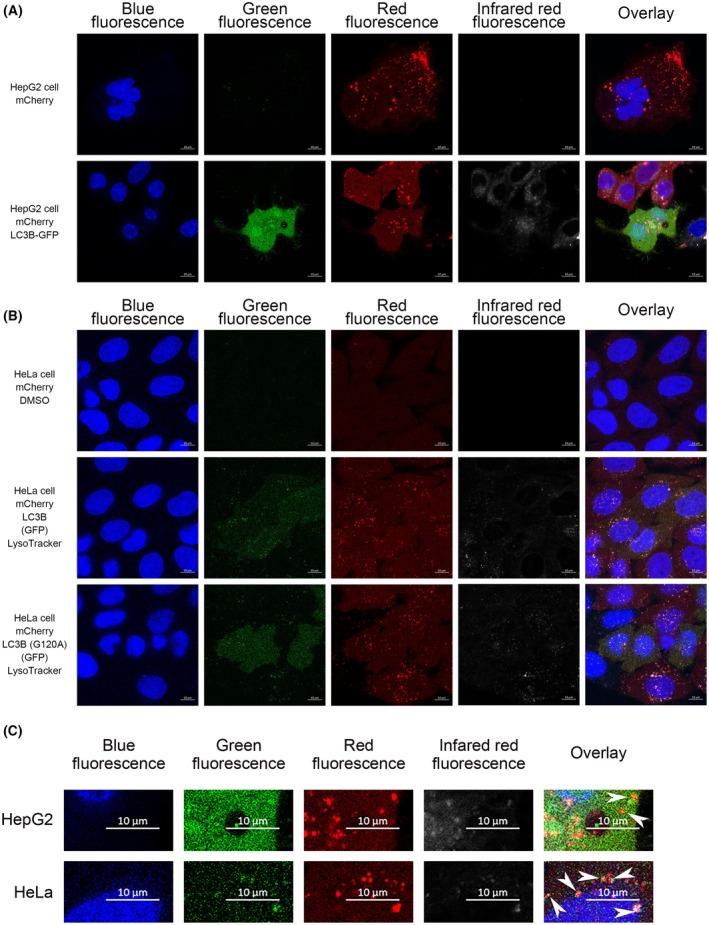
Colocalization of mCherry and LC3B, a marker of autophagosome. Colocalization of mCherry with autophagosome (LC3B) and lysosome in HepG2 (A) and HeLa (B) cells. (C) Enlarged image of insects of A and B. Arrowheads indicate colocalized granules of mCherry and LC3B‐GFP. DMSO: dimethyl sulfoxide, CQ: 100 μmol·L^−1^ chloroquine diphosphate. The magnification of the objective lens was 100×. Scale bar, 10 μm.

**Fig. 4:**
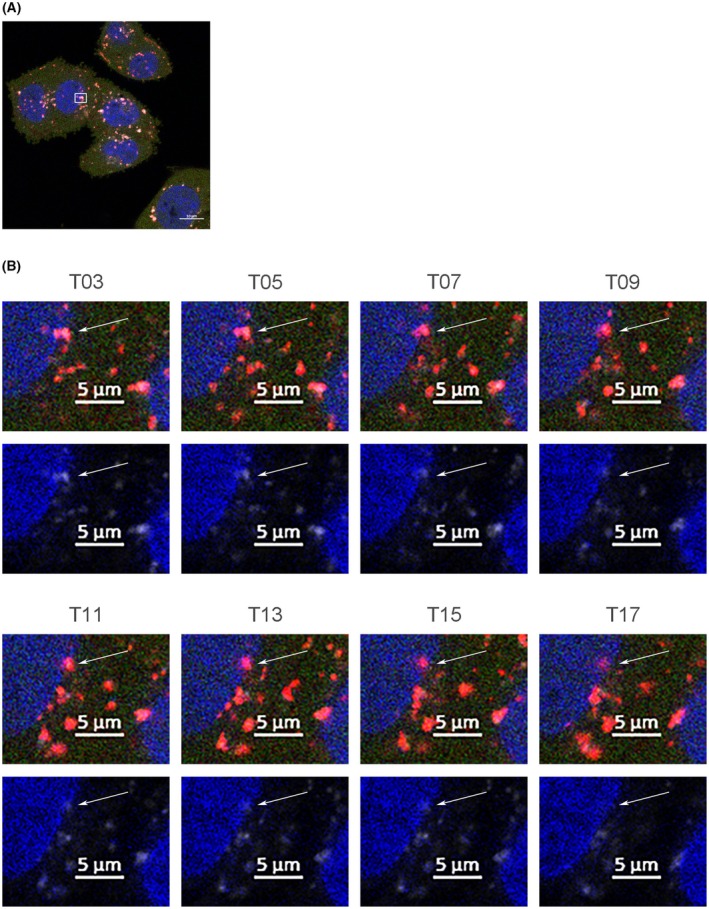
Extracted time‐lapse images (part of Videos S1 and S2) of HeLa cells transfected with the pTandem (mCherry/EGFP) Puro plasmid and stained with LysoTracker Deep Red (A). The time course of changes in full colour images and images without fluorescent proteins in the image field (inset of A) is in (B). Arrows indicate the fluorescent granules that directly entered the nucleus (upper image), and the image of the corresponding granules stained with LysoTracker Deep Red (lower image) in HeLa cells is also shown. The magnification of the objective lens was 100×. Scale bar, 5 μm.

**Fig. 5:**
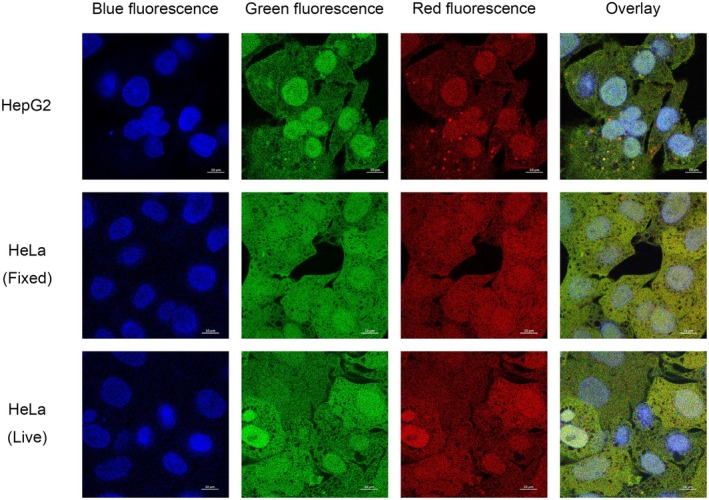
Fluorescent images of HepG2 and HeLa cells transfected with the pTandem Puro plasmid. Upper and middle images are HepG2 and HeLa cells, respectively, fixed with 4% PFA. Image of HepG2 cells was stored in 0.1 mol·L^−1^ PB for 4 days and that of HeLa cells for 3 days after fixation. Lower images are acquired by live cell imaging of HeLa cells transfected with pTandem Puro plasmid. Magnification of objective lens is ×100. Scale bar = 10 μm.

Laser confocal microscopy revealed the accumulation of both mCherry and mCherry/EGFP tandem proteins in HeLa cell nuclei (Figs 1, 2, 3). Cotranslocation analysis of mCherry in the nuclei, autophagosomes, and lysosomes revealed the colocalization of mCherry fluorescence in HeLa cell nuclei and lysosomes (Fig. S3). Moreover, mCherry and autophagosomes were partially colocalized in HeLa cells (Fig. S3). The fluorescence threshold of each fluorescent agent (white crossbar) was determined in HeLa cells without transfection or staining (data not shown). mCherry tracking revealed that mCherry was localized in the autolysosomes before entering the nucleus in HeLa cells (Figs 1 and 2). Furthermore, mCherry colocalized with Hoechst 33342 and LysoTracker Deep Red in HeLa cells, with some granules showing co‐localization of mCherry and DAPGreen (Fig. 1; Fig. S3). Notably, the addition of DMSO instead of DAPGreen—Autophagy Detection and LysoTracker Deep Red did not affect the translocation of fluorescent granules (Fig. 1). To confirm colocalization of autophagosome and FPs, LC3B, a marker of autophagosome colocalized with puncta of mCherry in both HepG2 and HeLa cells transfected with pmCherry Puro plasmid as well as DAPGreen (Fig. 3), indicating that mCherry could be degraded via the autophagy in both HepG2 and HeLa cells. In addition, On the contrary, fluorescent of LC3B (G120A)‐EmGFP distributed in whole cell of HeLa cells overexpressing mCherry, including nucleus as well as mCherry, that is, LC3B (G120A)‐EmGFP may be degraded and transported into nuclei as well as mCherry, whereas green fluorescence of LC3B‐GFP in cell nucleus of HeLa cell was less than that of LC3B (G120A)‐EmGFP (Fig. 3B). Lysosomes containing tandem FPs, which were identified via live cell imaging (e.g., mCherry/EGFP), entered the HeLa cell nuclei, and red fluorescence intensity did not decrease until the lysosomes entered the nuclei of HepG2 cells and HeLa cells (Fig. 4; Videos S1– S3). Green fluorescence of EGFP in tandem FPs in HeLa cells was temporarily quenched in the lysosome colocalized granules and recovered after the red fluorescent granules entered the HeLa cell nuclei (Videos S1 and S2). These results suggest that the lysosomes are successfully acidified in such cells, as the fluorescence of EGFP decreases under acidic conditions (Figs 2, 4) [28, 32]. So, we confirmed whether EGFP is completely degraded in lysosome in such cells. In fixed HepG2 cells expressing the tandem FPs, there were the granules where green fluorescence was absent (Fig. 5, upper panel). On the contrary, in fixed HeLa cells expressing tandem FPs (Fig. 5, middle panel), green fluorescence was recovered but green fluorescence was absent in lysosomes of live HeLa cells (Fig. 5, red dots of lower panel), indicating that 3 days of treatment of 0.1 mol·L^−1^ PB containing 0.1% PFA is enough to make intralysosomal pH to neutral and EGFP cannot be fully degraded as in fixed HepG2 cells and live HeLa cells (Fig. 5).

### The effects of pharmacological inhibition and knockdown of STX17 on nuclear translocalization of mCherry

For the first time, simultaneous comparison of intensity of nuclear mCherry between HepG2 and HeLa cells revealed that accumulation of mCherry in nuclei of HeLa cells was more than that of HepG2 cells (about 1.7‐fold of HepG2 cells, *P* < 0.01 vs. fluorescent intensity of nuclear mCherry in HepG2 cells, Fig. S4).

**Fig. 6:**
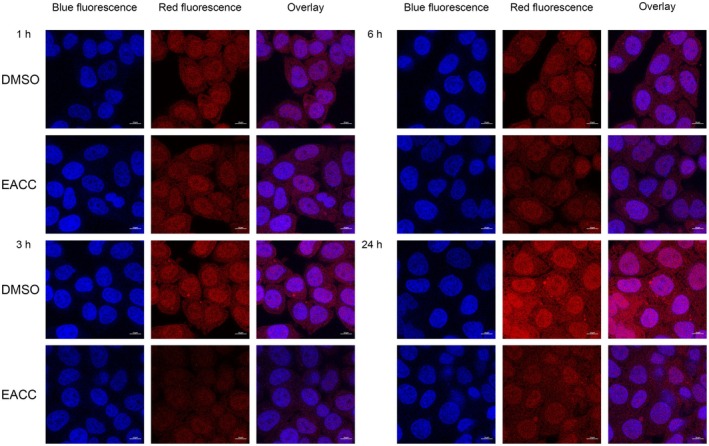
Time‐dependent inhibition of mCherry nuclear translocation by ethyl (2‐[5‐nitrothiophene‐2‐carboxamido] thiophene‐3‐carbonyl) carbamate in HeLa cells. Magnification of objective lens is ×100. Scale bar = 10 μm.

**Fig. 7:**
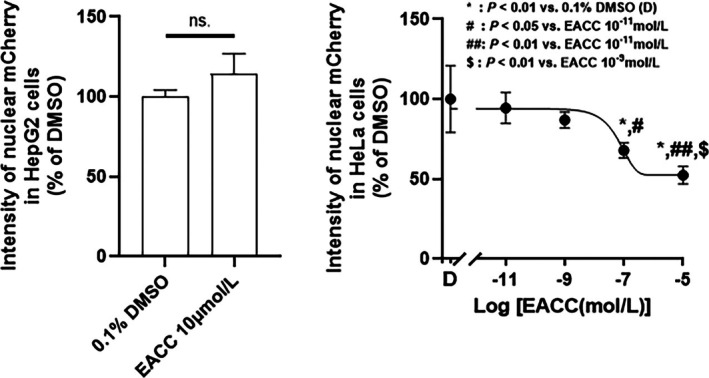
Quantitative analysis of dose‐dependent effects of ethyl (2‐[5‐nitrothiophene‐2‐carboxamido] thiophene‐3‐carbonyl) carbamate on HepG2 cells and HeLa cells at 3 h. Data were represented as mean (SD) and *P* values were calculated by Dunnett's test. *P < 0.01 vs. 0.1% DMSO (D), ^#^ *P* < 0.05 vs. EACC 10^−11^ mol·L^−1^, ^##^ *P* < 0.01 vs. EACC 10^−11^ mol·L^−1^. ^$^ *P* < 0.01 vs. EACC 10^−9^ mol·L^−1^.

Intensity of nuclear red fluorescence in HepG2 cells may not be clearly changed by EACC (Fig. S5), while EACC markedly decreased that the mCherry fluorescence intensity in the perinuclear lesions of HeLa cells and its nuclear translocation after 3 h (Fig. 6). Quantitative analysis at 3 h revealed that intensity of mCherry in HepG2 cells was not significantly decreased and EACC (1 μmol·L^−1^ and 10 μmol·L^−1^) significantly suppressed nuclear translocation of mCherry in HeLa cells dose‐dependently (*P* < 0.01 (EACC 1 μmol·L^−1^, 68.0 (4.6) % intensity of 0.1% DMSO, and 52.5 (5.5) % intensity of 0.1% DMSO, EACC 10 μmol·L^−1^) vs. 0.1% DMSO, Figs 7, S6). DMSO (0.1%) did not affect intensity of nuclear mCherry in both HepG2 and HeLa cells (Fig. S7).

**Fig. 8:**
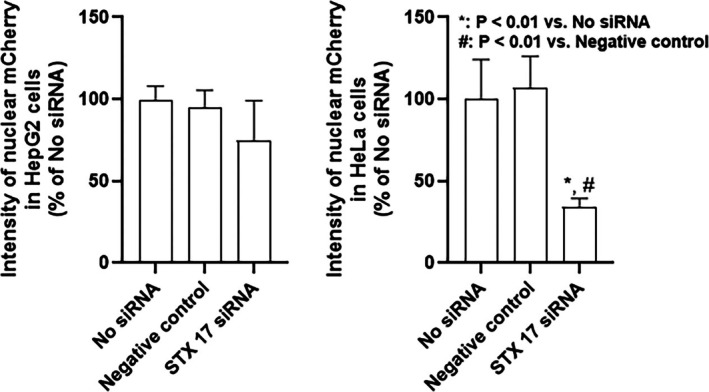
Bar graphs of intensity of nuclear mCherry transfected with or without STX17 siRNA in HepG2 and HeLa cells. Data were represented as mean (SD) and *P* values were calculated by Dunnett's test. *P < 0.01 vs. the cells without transfection of STX17 siRNA.

In addition to pharmacological inhibition of syntaxin 17, inhibition of fusion of autophagosome with lysosome was performed by knocking down STX 17 to exclude nonspecific effects of EACC. RNA interference of STX17 also significantly decreased nuclear translocation of mCherry in HeLa cells transfected with pmCherry Puro plasmid (34.1 (5.4) % nuclear intensity of mCherry in no transfection group, *P* < 0.01). Transfection of negative control siRNA (106 (19.5) % nuclear intensity of mCherry in no transfection group) did not change nuclear intensity of mCherry in HeLa cells, but not in HepG2 cells (negative control group: 89.5 (16.3) % nuclear intensity of mCherry in no transfection group, and STX17 siRNA group: 74.6 (24.5) % nuclear intensity of mCherry in no transfection group, Figs 8, S8).

### Autolysosome and nucleus in HepG2 and HeLa cells: The study using TEM

**Fig. 9:**
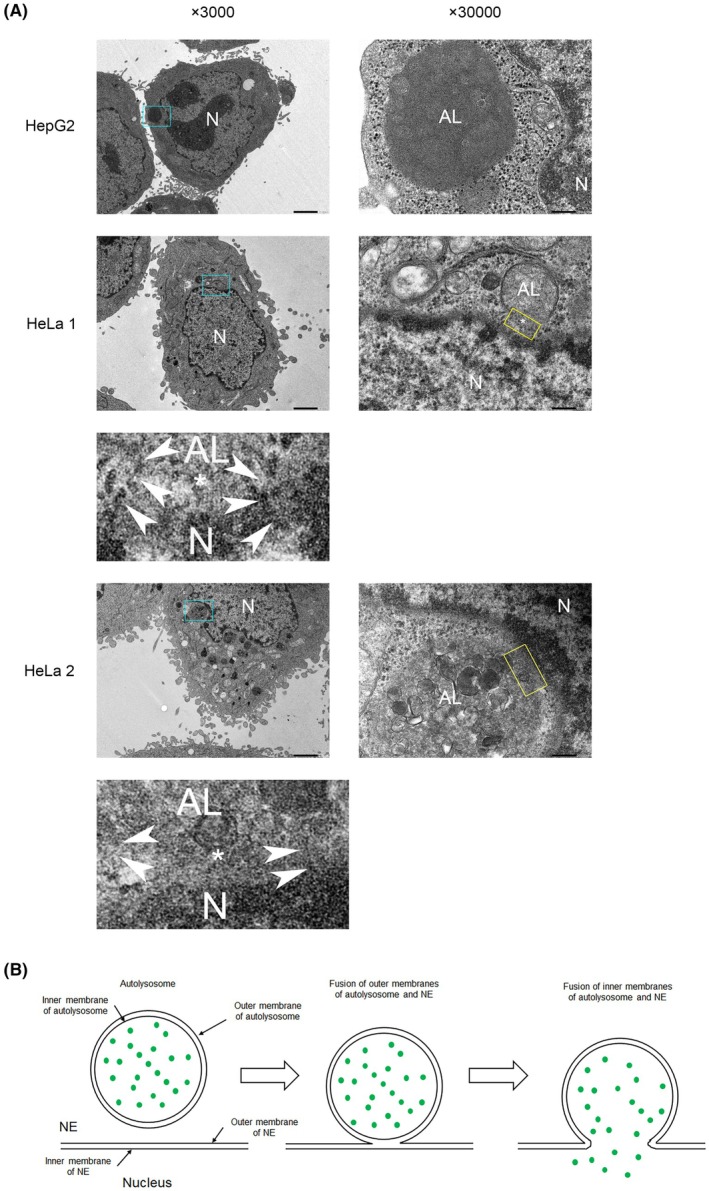
Typical image of HepG2 and HeLa cells transfected with pmCherry Puro plasmid. (A) Electron microscopic images of HepG2 and HeLa cells transfected with pmCherry Puro plasmid. Magnitude of a left panel is ×3000 and that of a right panel is ×30 000 (blue insect in left panel). The image in lower panels of HeLa cells transfected with pmCherry Puro plasmid 1/2 is the closed up image of the yellow insect in the upper right panel (×30 000 image). Arrow heads indicate the possible fusion point of autolysosome and nucleus. Asterisk indicates orifice of autolysosome to nucleus. AL: Autolysosome; N: Nucleus; Scale bars indicate 2.0 μm for image of ×3000 and 200 nm for that of ×30 000. (B) The possible mechanism of fusion of autolysosome and NE.

TEM revealed that no lysosome or autolysosome fused to the nucleus in HepG2 cells transfected with pmCherry Puro plasmid (HepG2, Fig. 9A). But the double membrane of the autolysosome of HeLa cells continued to those of the nuclear envelope (NE, HeLa 1/2, arrow heads in right panels, Fig. 9A), indicating that an autolysosome can fused cell nucleus in HeLa cells transfected with pmCherry Puro plasmid, and the possible mechanism of membrane fusion of autolysosome and the NE is shown in Fig. 9B.

### Intracellular localization of tandem FP in HepG2 cells

**Fig. 10:**
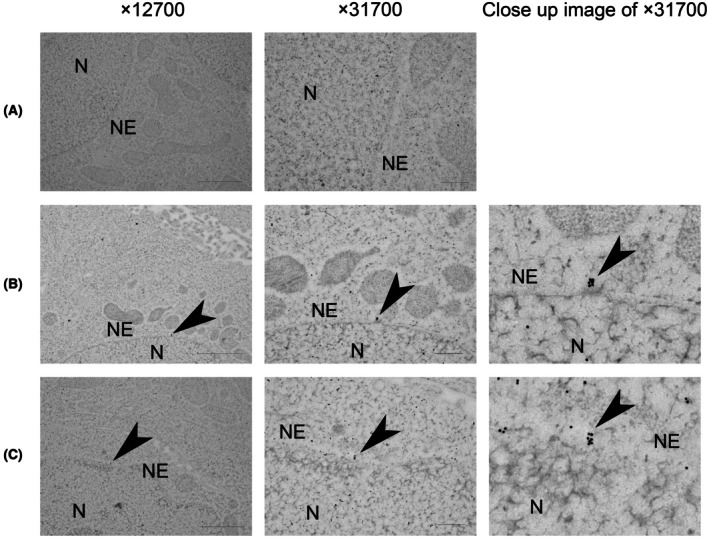
Images of ITEM of EGFP in HepG2 cells transfected with or without pTandem plasmid labelled with anti‐GFP antibody. Arrow heads indicate immunoreactivity of EGFP. (A) Images (a left panel: ×12 700 and a right panel: ×31 700) of ITEM of HepG2 cells without transfection. (B and C) Images (left panels: ×12 700 and middle panels: ×31 700) of ITEM of HepG2 cells transfected with pTandem Puro plasmid. Right panels represent digitally close‐up image of the middle panels. N: nucleus, NE: nuclear envelope, NPC: nuclear pore complex. Scale bars indicated 2 μm in left panels and 500 nm in a right panel of A and middle panels of B and C, respectively.

Immunoreactivity of GFP was not found in HepG2 cells without transfection (Fig. 10A), indicating that there were no nonspecific findings in this study. Immunoreactivities of EGFP were detected in the cytosol and nucleus (Fig. 10B,C). And those were also detected in the NPCs of HepG2 cells transfected with pTandem Puro plasmid, indicating that tandem FP can be translocalized to the nucleus by passing through the NPCs of HepG2 cells.

## Discussion

Several receptor ligands translocalize to the nucleus [1, 2, 3, 4, 5, 6, 7, 8, 9, 10, 11, 12, 33]; however, the detailed mechanisms remain ambiguous. On the way to the study on the nuclear translocalization of UCN I, a ligand of CRF_1_ and CRF_2_ [13], we found the FPs were accumulated in the nucleus of HeLa cells, in which UCN I was translocalized to cell nucleus, and hypothesized that the common mechanism(s) of nuclear translocalization of these peptides may be existed. Therefore, the present study was designed to elucidate the nuclear translocation mechanisms of FPs in this study. HeLa cells were transfected with FP‐expressing plasmids. The FPs were translocalized to the cell nuclei, we guessed that the nuclear translocation mechanisms of FPs involve the autophagosome–lysosome system, as protein aggregates may be degraded by autophagy [17, 18]. Alternatively, because macromolecules of which molecular weight is under 60 kDa can be transported to cell nucleus [14], we guessed that FPs, which lacks known NLS [31], may be also transported to cell nucleus via the NPCs. In autophagy, a degradation process classified into aggrephagy, lysophagy, mitophagy, macroautophagy, chaperone‐mediated autophagy, RN/DNautophagy, and microautophagy, degradative enzymes are acquired via fusion with the lysosome [17, 34]. Here, the fluorescence of mCherry overlapped with that of the nuclei in HeLa cells, indicating an atypical transport system in HeLa cells. The green fluorescence of DAPGreen, an autophagosome marker representing the location of autophagosome [24], also overlapped the red fluorescence of granules containing mCherry. And colocalization of mCherry and LC3B, a marker of autophagosome [25, 26, 27], confirmed that autophagosome may be involved in degradation of FPs as an entry of autophagosome–lysosome degradation system. We also observed that LysoTracker Deep Red‐labelled granules containing tandem FP were turned to red (i.e., red‐fluorescent granules in Fig. 2 and Video S1; because pH of the lysosome usually is acidic [35] and fluorescence of EGFP decreases under acidic conditions [28, 32]) and directly entering the cell nuclei. Green fluorescence of EGFP of tandem FP was recovered in lysosome of HeLa cells, but not in that of HepG2 cells, indicating that fluorescence of EGFP in tandem FP could be degraded in lysosome faster than mCherry and this result is corresponding to previous results [36, 37]. Together with these results, FPs can be incorporated into phagophore, and, in turn, autolysosome may fuse with NE. Thereafter, inner membrane of autolysosome opens and released the substance in autolysosome into nucleus in HeLa cells. On the contrary, intact tandem FP could localized to lysosome in HeLa cells stored in 0.1 mol·L^−1^ PB containing 0.1% PFA for 3 days, indicating that it is enough time to elevate intralysosomal pH to neutral because the recovery of intralysosomal green fluorescence in HeLa cells stored in 0.1 mol·L^−1^ PB containing 0.1% PFA for 3 days. But intralysosomal green fluorescence in HepG2 cells stored in 0.1 mol/L PB containing 0.1% PFA for 4 days after fixation with 4% PFA was not recovered, indicating that EGFP may be normally degraded in lysosome of HepG2 cells. These results are corresponding to previous one and indicated that FPs may be transported to nuclei via the transport route other than the fusion of nuclei and autolysosome, for example, via the NPC in HepG2 cells, because it was reported that threshold of molecular weight of nuclear translocalization of protein aggregates without nuclear localization signal via the NPC is 30–60 kDa [14] and molecular weight of mCherry and tandem FP were 27 kDa and 55 kDa, respectively. The results indicated that autolysosomes containing mCherry entered the nucleus after autophagosome fusion with lysosomes. To confirm this idea, we examined whether the nuclear fluorescence of mCherry was decreased by EACC, an inhibitor of autophagosome–lysosome fusion that affects the translocation of the soluble N‐ethylmaleimide‐sensitive factor attachment protein receptors, syntaxin 17, and soluble N‐ethylmaleimide‐sensitive factor attachment protein 29, on autophagosomes without impeding their functions [29]. And to confirm the role of autophagosome–lysosome fusion in nuclear translocalization of mCherry by knockdown of syntaxin 17, a key molecule in autophagosome–lysosome fusion [30], we check the role of syntaxin 17 by knockdown of syntaxin 17 in addition to pharmacological inhibition of syntaxin 17 with EACC. For the first time, we checked the difference of nuclear mCherry expression between HepG2 and HeLa cells used in this study. As a result, mCherry was localized more intense in HeLa cells than HepG2 cells. In addition, knocking down of syntaxin 17 RNA also resulted in decrease in nuclear translocalization of mCherry. These results indicated that fusion of autophagosome with lysosome by syntaxin 17 could be involved in the nuclear translocalization process of FPs into nuclei involves autophagy in HeLa cells and nuclear mCherry may be rapidly degraded in nucleus. On the contrary, in HepG2 cells, pharmacological inhibition and knockdown of syntaxin 17 did not exert significant action on nuclear translocalization of mCherry, indicating that FPs could be transported into cell nuclei via the transportation system other than autophagosome–lysosome system. Therefore, these results suggest two feasibilities in the nuclear translocation of FPs: (1) FPs are directly transported into the nucleus via NPCs because the molecular weight of FPs facilitates their passing through the NPC [14] in both HepG2 and HeLa cells and/or (2) autolysosomes containing FPs directly fuse to the nucleus and FPs not degraded in the lysosome are released into the nucleus of HeLa cells, but unlikely into that of HepG2 cells.

To confirm the hypothesis that autolysosome fuses with nucleus (or nuclear envelope), we performed the TEM experiments using HeLa cells transfected with mCherry. TEM images of HeLa cells overexpressing mCherry revealed that autolysosome and NE fused and autolysosome opened to nucleus in HeLa cells, but not HepG2 cells. Autophagosome can fuse with NE because both autophagosome and NE consisted of double membrane [38, 39], that is, outer membranes and, in turn, inner membranes of autophagosome and nuclear membrane can fuse and open to the nucleus. Usually, after autophagosome fused with lysosome, inner membrane of autophagosome is degraded but inner membrane of autophagosome in HeLa cells remained. The tracking experiments of tandem fluorescent protein accompanied with in HepG2 and HeLa cells revealed autolysosome in HeLa cells revealed that tandem FPs in HeLa cells was degraded insufficiently, whereas tandem FPs may be completely degraded in HepG2 cells, indicating that the inner membrane of autophagosome in HeLa cells cannot be degraded because the substances in autophagosome in HeLa cells may be degraded incompletely. And our results that autophagosome–lysosome mechanism can be involved in intracellular transport of receptor ligands into nucleus, is leading to the hypothesis that angiotensin II in cardiomyocytes under high glucose condition [3] may be caused by disruption of lysosome function because high glucose can affect lysosome function [40]. Although, to the best of our knowledge, we do not know whether the action of nuclear localization of receptor ligands or endogenous substances on cellular function is beneficial or harmful, our present data may indicate that nuclear translocalization of such substances can indicate a certain pathological condition.

NPCs, where the vast majority of material exchange occurs in the nuclear envelope [16], are another candidate route of nucleus to nucleus transport because the molecular size of FPs used in the present study is less than 60 kDa [14]. Our results revealed that the tandem FP, of which the molecular weight is 55 kDa, was localized in NPCs of HepG2 cells, indicating that FPs can be transported into the nucleus through NPCs. But our present study cannot reveal whether the nuclear transport receptor(s), for example, importins [41], are involved in the nuclear translocalization of FPs through NPCs.

In conclusion, our results demonstrated that the entry of autolysosomes, which fused with NE and into the nucleus was involved in the nuclear translocation of FPs in HeLa cells which are positive for immunoreactivity of a receptor ligand (e.g., UCN I), suggesting that lysosomal behaviour also causes the nuclear translocation of receptor ligands, as lysosomes are involved in the degradation of late endosomes. Therefore, entry of lysosomes into the nucleus possibly occurs via degradation systems for other proteins or peptides. Our present findings suggest cell nuclei (as in the case of HeLa cells) can be involved as the degradation region for numerous endogenous proteins. However, further studies are necessary to validate these findings.

## Conflict of interest

The author declares no conflict of interest.

## Author contributions

KI designed the project, acquired images, interpreted the data, and wrote the manuscript.

## Supporting information


**Fig. S1.** mCherry and AcGFP fluorescent proteins localized in the HeLa cell nuclei.
**Fig. S2.** Western blot analysis on molecular size of expressed FPs in HepG2 and HeLa cells.
**Fig. S3.** Typical images of the colocalization of mCherry with Hoechst 33342 (nucleus), DAPGreen (autophagosome), and LysoTracker Deep Red (lysosome) in HeLa cells stably expressing mCherry.
**Fig. S4.** Comparison of intensities of nuclear mCherry in HepG2 and HeLa cells.
**Fig. S5.** Time‐dependent inhibition of mCherry nuclear translocation by EACC in HepG2 cells transfected with pmCherry Puro plasmid.
**Fig. S6.** Typical image of dose‐dependent effects of EACC in HepG2 cells and HeLa cells at 3 h.
**Fig. S7.** The effects of STX17 knockdown on the nuclear translocalization in HepG2 and HeLa cells transfected with pmCherry Puro plasmid.
**Fig. S8.** The effects of dimethysulfoxide (DMSO) on nuclear localization of mCherry in HepG2 and HeLa cells.

**Video S1.** Time‐lapse images of HeLa cells transfected with the pTandem (mCherry/EGFP) plasmid and stained with LysoTracker Deep Red (full colour).

**Video S2.** Time‐lapse images of HeLa cells in the same sample shown in Video S1 (fluorescence of LysoTracker Deep Red [Cy5] and Hoechst 33342).

**Video S3.** Time‐lapse images of HepG2 cells transfected with the pTandem (mCherry/EGFP) plasmid and stained with LysoTracker Deep Red (full colour).

## Data Availability

Vector sequences and maps used in this study are available at www.vectorbuilder.com. And all image data are available from the published article.
