# Supplementary material for: Evaluating the involvement of autolysosomes in the nuclear translocation of fluorescent proteins
Source: FEBS Open Bio. 2026 Jun 3:10.1002/2211-5463.70279. Online ahead of print. doi: 10.1002/2211-5463.70279 (PMC13399571; doi:10.1002/2211-5463.70279)
Supplement: Supplementary file 1 — Fig. S1. mCherry and AcGFP fluorescent proteins localized in the HeLa cell nuclei. Fig. S2. Western blot analysis on molecular size of expressed FPs in HepG2 and HeLa cells. Fig. S3. Typical images of the colocalization of mCherry with Hoechst 33342 (nucleus), DAPGreen (autophagosome), and LysoTracker Deep Red (lysosome) in HeLa cells stably expressing mCherry. Fig. S4. Comparison of intensities of nuclear mCherry in HepG2 and HeLa cells. Fig. S5. Time‐dependent inhibition of mCherry nuclear translocation by EACC in HepG2 cells transfected with pmCherry Puro plasmid. Fig. S6. Typical image of dose‐dependent effects of EACC in HepG2 cells and HeLa cells at 3 h. Fig. S7. The effects of STX17 knockdown on the nuclear translocalization in HepG2 and HeLa cells transfected with pmCherry Puro plasmid. Fig. S8. The effects of dimethysulfoxide (DMSO) on nuclear localization of mCherry in HepG2 and HeLa cells. [file FEB4-9999-0-s003.pdf]

Supporting Information

Evaluating the involvement of autolysosomes in the nuclear translocation of fluorescent proteins

Keiichi Ikeda

Core Research Facilities for Basic Science, Research Center for Medical Sciences, The Jikei University School of Medicine, 3-25-8, Nishishimbashi, Minato-ku, Tokyo 105-8461, Japan

Content

Supplemental Figure and Figure legends 3

Supplemental video legends 12

Supplementary Figures and Figure Legends

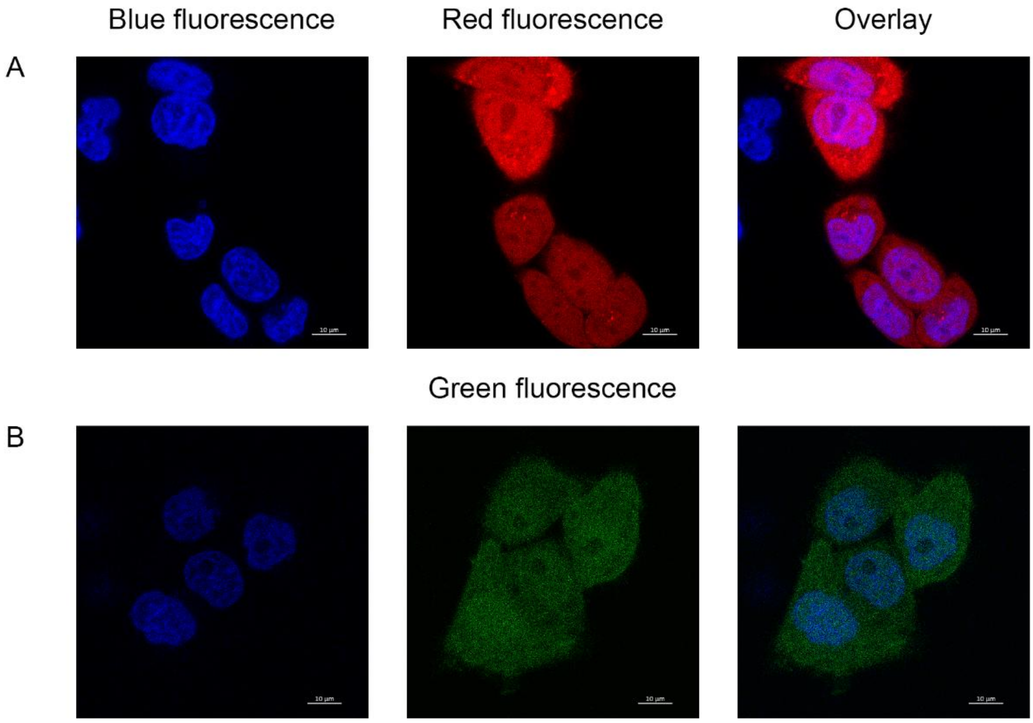

Fig. S1. mCherry and AcGFP fluorescent proteins located in the nuclei of HeLa cells. A. Laser confocal microscopy image of pmCherry-C1 plasmid-transfected HeLa cells. B. Confocal laser microscopy image of pAcGFP-C1 plasmid-transfected HeLa cells. Magnification of the objective lens is ×100. Scale bar = 10 μm.

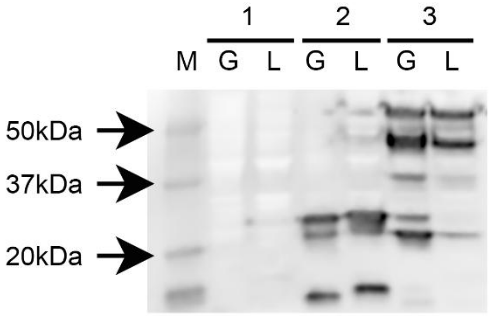

Fig. S2. Western blot analysis on molecular size of expressed FPs in HepG2 and HeLa cells. Western blot analysis on mCherry and Tandem FP expressed in HepG2 (G) and HeLa (L) cells. M: Protein marker. 1: HepG2 and HeLa cells without transfection. 2: HepG2 and HeLa cells transfected with pmCherry Puro plasmid. 3: HepG2 and HeLa cells transfected with pTandem Puro plasmid.

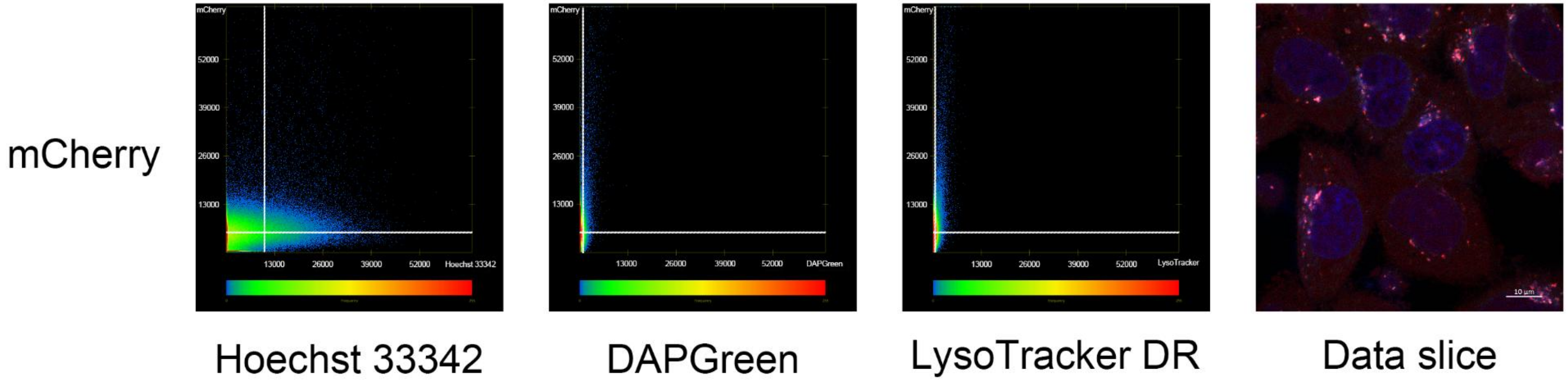

Fig. S3. Typical images showing colocalisation of mCherry and Hoechst 33342 (nucleus), DAPGreen (autophagosome), or LysoTracker Deep Red (lysosome) in HeLa cells stably expressing mCherry. The threshold for each colour was determined by fluorescence in HepG2 and HeLa cells without transfection (data not shown). The right-sided image represents the colocalisation analysis. White cross bars indicate the threshold of fluorescence determined for cells without transfection, and the colour bars under the graphs indicate the number of granules. Magnification of objective lens is ×100. Scale bar = 10 μm.

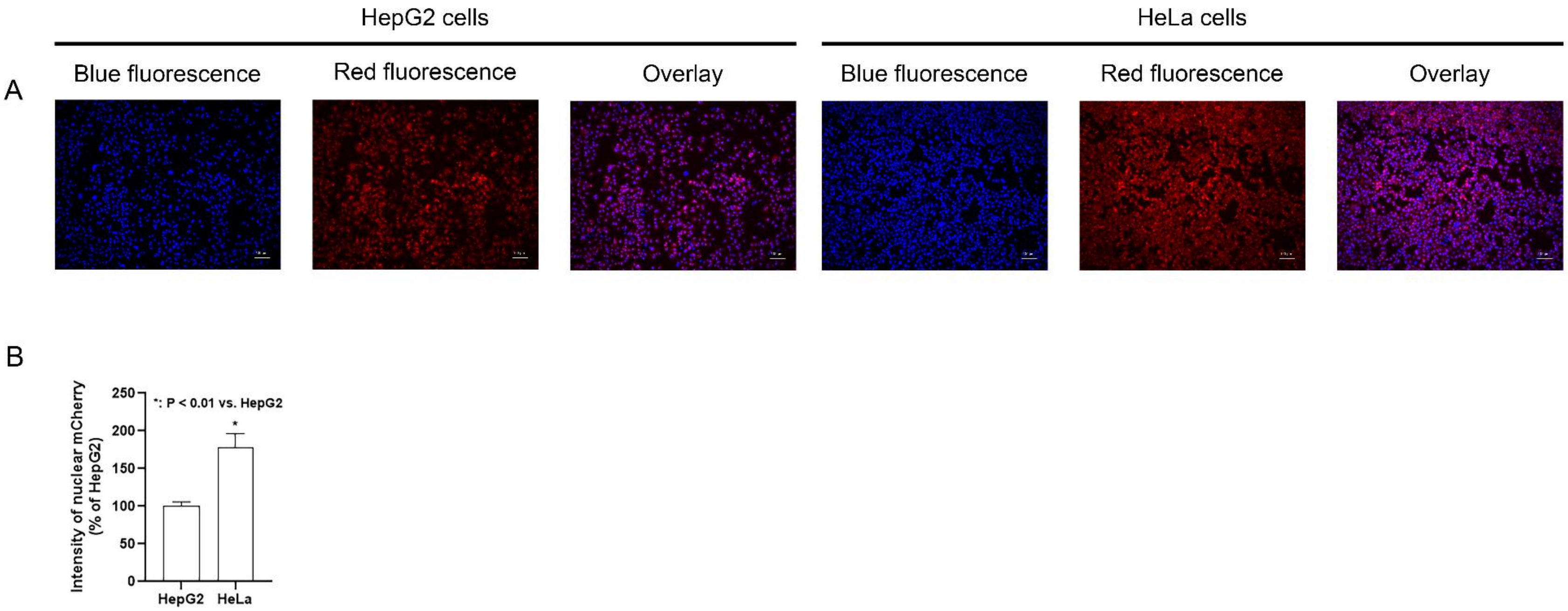

Fig. S4. Comparison of intensities of nuclear mCherry in HepG2 and HeLa cells. A: Typical images of mCherry intensity in HepG2 and HeLa cells transfected with pmCherry Puro plasmid. B: A bar graph of nuclear intensity of mCherry (unpaired student *t* test [GraphPad Prism 10, GraphPad software LLC, Boston, MA, USA], colocalised blue and red fluorescence in each cell). Data were represented as mean (SD). Magnification of objective lens is ×10. Scale bar = 100 μm.

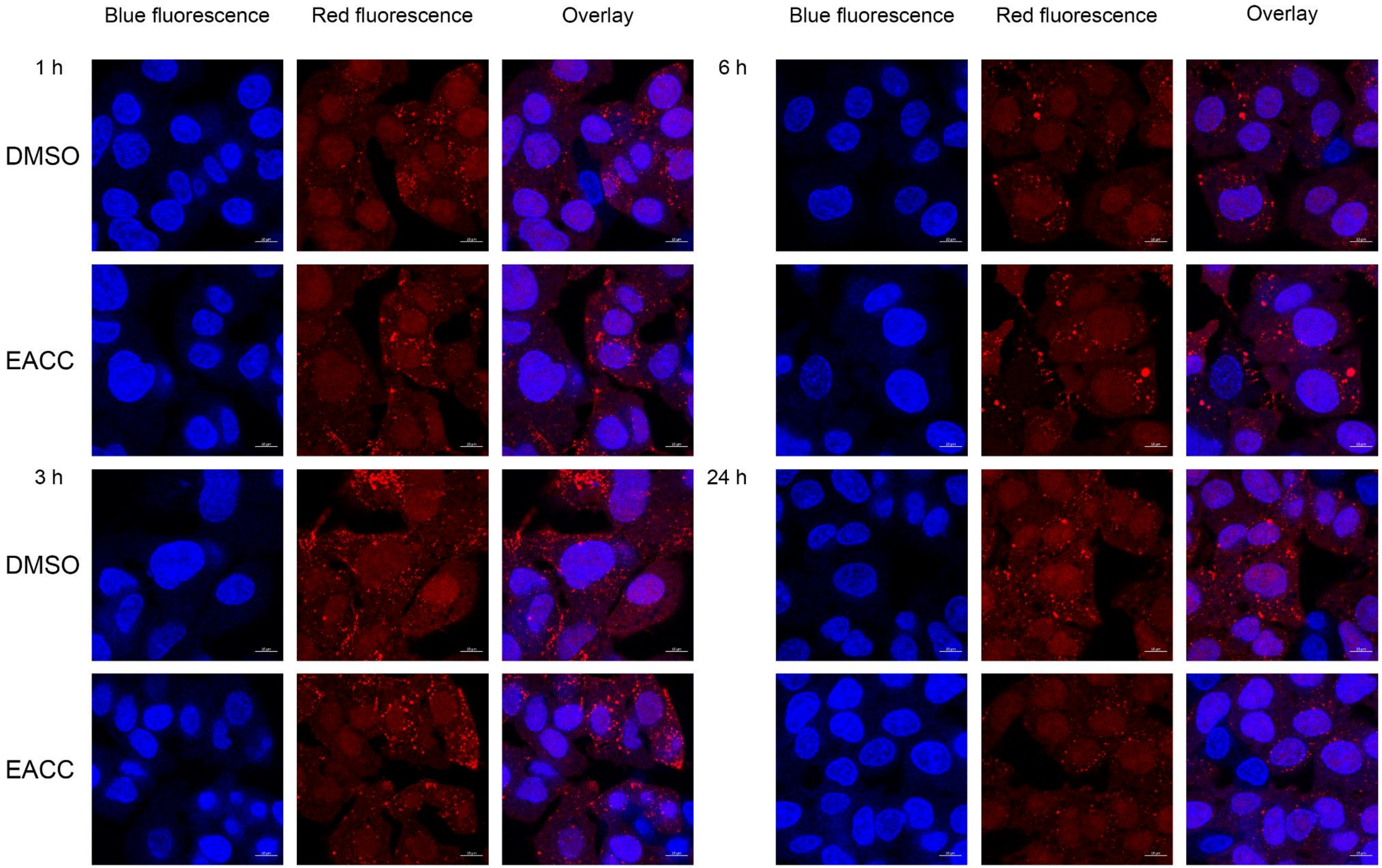
Blue fluorescence
Red fluorescence
Overlay
1 h
6 h
3 h
24 h
DMSO
EACC
DMSO
EACC

Fig. S5 Time-dependent inhibition of mCherry nuclear translocation by ethyl (2-[5-nitrothiophene-2-carboxamido] thiophene-3-carbonyl) carbamate in HepG2 cells. Magnification of objective lens is ×100. Scale bar = 10 μm.

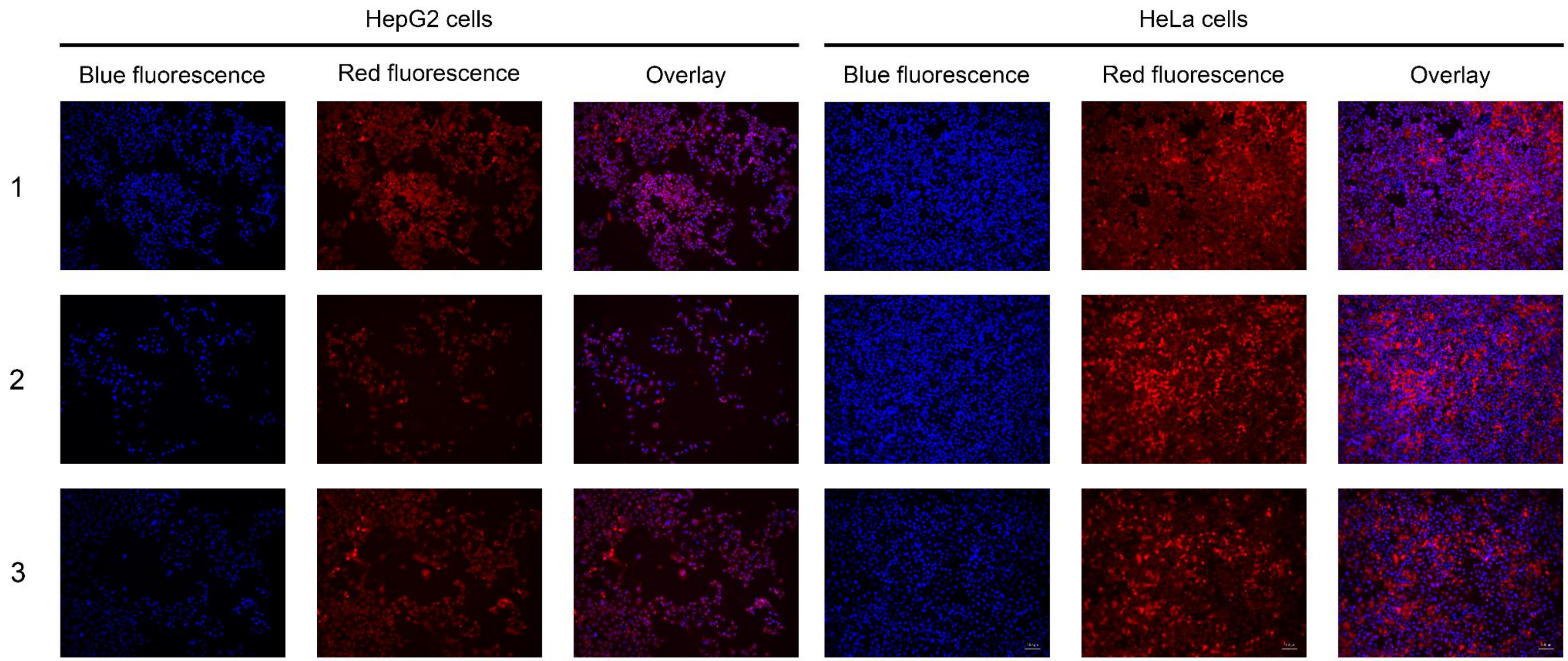

Fig. S8. Images of HepG2 and HeLa cells transfected with pmCherry Puro plasmid and STX17 siRNA. 1: no siRNA, 2: Negative control siRNA, 3: STX17 siRNA. Magnitude of objective lens was ×10. Scale bar = 100 μm.

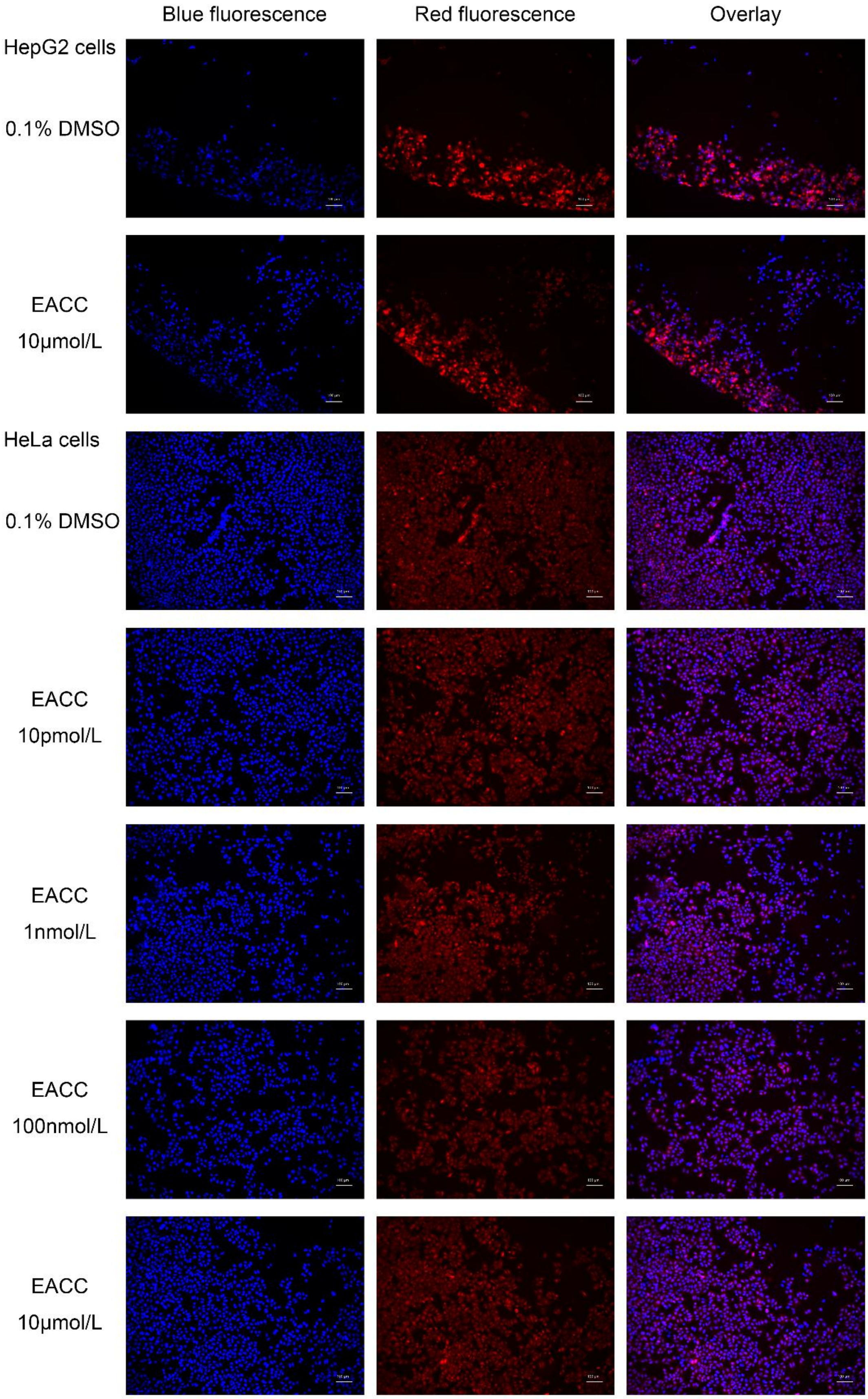
Blue fluorescence
Red fluorescence
Overlay
HepG2 cells
0.1% DMSO
EACC
10μmol/L
HeLa cells
0.1% DMSO
EACC
10pmol/L
EACC
1nmol/L
EACC
100nmol/L
EACC
10μmol/L

Fig. S6 Typical image of dose-dependent effects of EACC in HepG2 cells and HeLa cells at 3 hours. HepG2 and HeLa cells transfected with pmCherry Puro plasmid were treated with 0.1% DMSO for 3 hours and HeLa cells transfected with pmCherry Puro plasmid were treated with 10pmol/L to 10μmol/L EACC for 3 hours. Magnitude of objective lens was ×10. Scale bar = 100 μm.

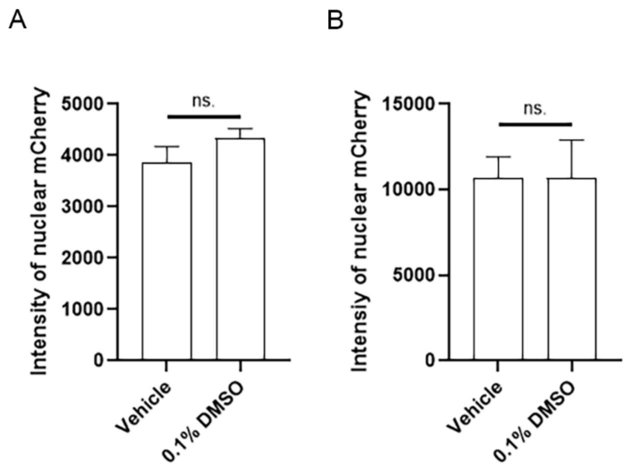

Fig. S7 The effects of DMSO on nuclear localisation of mCherry in HepG2 (A) and HeLa (B) cells. Data were represented as mean (SD) and statistical analysis was performed by unpaired student *t* test.

Supplementary video 1: Time-lapse image of HeLa cells transfected with the pTandem (mCherry/EGFP) plasmid and stained with LysoTracker Deep Red (full colour). Magnification of objective lens is ×100. Scale bar = 10 μm.

Supplementary video 2: Time-lapse image of HeLa cells in the same sample as Supplementary video 1 (fluorescence of LysoTracker Deep Red [Cy5] and Hoechst 33342). Magnification of objective lens is ×100. Scale bar = 10 μm.

Supplementary video 3: Time-lapse image of HepG2 cells transfected with the pTandem (mCherry/EGFP) plasmid and stained with LysoTracker Deep Red (full colour). Magnification of objective lens is ×100. Scale bar = 10 μm.
